# Mesenchymal Stromal Cells Support Endometriotic Stromal Cells *In Vitro*

**DOI:** 10.1155/2018/7318513

**Published:** 2018-01-28

**Authors:** Fawaz Abomaray, Sebastian Gidlöf, Bartosz Bezubik, Mikael Engman, Cecilia Götherström

**Affiliations:** ^1^Division of Obstetrics and Gynecology, Department of Clinical Science, Intervention and Technology, Karolinska Institutet, Stockholm, Sweden; ^2^Centre for Hematology and Regenerative Medicine, Karolinska Institutet, Stockholm, Sweden; ^3^Center for Fetal Medicine, Patient Area of Pregnancy and Childbirth, Karolinska University Hospital, Stockholm, Sweden; ^4^Department of Women's and Children's Health, Karolinska Institutet, Stockholm, Sweden; ^5^Department of Obstetrics and Gynecology, Karolinska University Hospital, Stockholm, Sweden; ^6^Department of Obstetrics and Gynecology, Danderyd's Hospital, Stockholm, Sweden

## Abstract

Endometriosis is an inflammatory disease marked by ectopic growth of endometrial cells. Mesenchymal stromal cells (MSC) have immunosuppressive properties that have been suggested as a treatment for inflammatory diseases. Therefore, the aim herein was to examine effects of allogeneic MSC on endometriosis-derived cells *in vitro* as a potential therapy for endometriosis. MSC from allogeneic adipose tissue (Ad-MSC) and stromal cells from endometrium (ESC_endo_) and endometriotic ovarian cysts (ESC_cyst_) from women with endometriosis were isolated. The effects of Ad-MSC on ESC_endo_ and ESC_cyst_ were investigated using *in vitro* proliferation, apoptosis, adhesion, tube formation, migration, and invasion assays. Ad-MSC significantly increased proliferation of ESC compared to untreated controls. Moreover, Ad-MSC significantly decreased apoptosis and increased survival of ESC. Ad-MSC significantly increased adhesion of ESC_endo_ and not ESC_cyst_ on fibronectin. Conditioned medium from cocultures of Ad-MSC and ESC significantly increased tube formation of human umbilical vein endothelial cells on matrigel. Ad-MSC may significantly increase migration of ESC_cyst_ and did not increase invasion of both cell types. The data suggest that allogeneic Ad-MSC should not be considered as a potential therapy for endometriosis, because they may support the pathology by maintaining and increasing growth of ectopic endometrial tissue.

## 1. Introduction

Endometriosis affects approximately 10% of women of reproductive age, is marked with ectopically growing endometrial cells, and exhibits increased local inflammation leading to chronic pelvic pain and infertility [1]. Despite medical and surgical treatments to reduce inflammation and remove ectopic lesions, recurrence or therapy resistance is very common [2]. Therefore, there is an urgent need of new therapies for endometriosis.

Despite active research, the pathogenesis of endometriosis remains largely unclear. The most commonly accepted theory is that endometriosis develops from reflux of menstrual debris into the pelvic cavity during menstruation, which then implants resulting in endometriosis [3]. Although almost all women exhibit retrograde menstruation, only approximately 10% develop endometriosis [4]. This conundrum must be explained by other factors playing a role in disease development [3, 4]. For example, the endometrium of women with endometriosis displays resistance to apoptosis with a subsequent increase in cell proliferation, migration, adhesion, and invasion of the mesothelial lining of the pelvic cavity and increased ability to induce angiogenesis to cause endometriosis [5].

The immunosuppressive properties of mesenchymal stromal cells, also called mesenchymal stem cells (MSC), have made them a potential treatment for inflammatory and autoimmune diseases such as graft versus host disease (GvHD), multiple sclerosis (MS), and Crohn's disease [6]. It has been suggested that the immunosuppressive properties of MSC are due to their ability to sense the changing levels of inflammation in their microenvironment and respond accordingly [7]. Therefore, MSC may be a potential therapy for the inflammatory component of endometriosis. More specifically, previously, it has been reported that allogeneic MSC derived from adipose tissue (Ad-MSC) have immunosuppressive properties with potential to treat inflammatory diseases such as GvHD and MS [8, 9]. Previously, it has been found that autologous MSC are altered by the pathology of endometriosis [10]. In addition, we found that MSC from the ectopic (ESC_cyst_) endometrium were phenotypically and functionally different from MSC from the eutopic (ESC_endo_) endometrium in women with endometriosis suggesting that autologous MSC may be altered by the pathology [11]. Therefore, in the present study, we aimed to investigate the effects of allogeneic Ad-MSC on endometriosis-derived cells *in vitro* as the first step of a long-term goal of developing a potential therapy for endometriosis. The effects of Ad-MSC on ESC_cyst_ and ESC_endo_ were examined using proliferation, apoptosis, adhesion, tube formation (*in vitro* angiogenesis), migration, and invasion assays, which are the aforementioned parameters that are perturbed in endometriosis. It was found that allogeneic Ad-MSC may promote ESC_cyst_ proliferation, survival, and migration and support ESC_cyst_ to promote tube formation of human umbilical vein endothelial cells (HUVEC) but did not affect adhesion or invasion of ESC_cyst_* in vitro*. The data suggest that allogeneic Ad-MSC should not be considered as a potential therapy for endometriosis because they may support the pathology by maintaining and increasing growth of ectopic endometrial tissue. Moreover, since MSC are present in ectopic lesions in endometriosis as confirmed by us [11] and others [12], this indicates that MSC are likely involved in the pathogenesis of endometriosis.

## 2. Materials and Methods

### 2.1. Human Tissue Samples

The inclusion criteria for the study were female women of fertile age suffering from endometriosis that have not undergone hormonal treatment for three to six months before undergoing laparoscopic surgery for confirmation of diagnosis and treatment. Three types of tissues were collected: (i) endometriotic ovarian cysts (ectopic endometrium) and (ii) endometrium (eutopic endometrium), which were both from women with endometriosis who underwent surgery for removal of endometriotic ovarian cysts, and (iii) adipose tissue from healthy women undergoing elective caesarean section at term. The endometriotic ovarian cysts and endometrium were collected from women aged 31 to 42 (36.3 ± 5.8 years (mean ± SD), *n* = 4) undergoing laparoscopic surgery for confirmation or treatment of endometriosis. All women were histologically confirmed to have endometriosis by a pathologist. Only one woman underwent hormonal treatment. Moreover, two of the biopsies were from the proliferative phase, one was unknown, and one had amenorrhea. The adipose tissue was collected from women aged 34 to 39 (36.5 ± 3.54 years (mean ± SD), *n* = 2). Informed oral and written consent was obtained from each participant, and ethical approval was obtained from The Regional Ethical Review Board in Stockholm (2013/1094-31/2, 2017/1017-32).

### 2.2. Isolation of Stromal Cells from Eutopic and Ectopic Endometrium

Human endometrial and endometriotic ovarian cyst tissues were digested to single cell suspension using 1 mg/mL collagenase type I (Sigma, Missouri, United States) diluted in Hank's Balanced Salt Solution (Life Technologies, Paisley, UK) (90 min for endometriotic tissue and 30 min for endometrial tissue) at 37°C with shaking every 10 min. The tissue digests were filtered twice through 100 *μ*m cell strainers (Corning, New York, United States), and eventually, the stromal cells were filtered through a 40 *μ*m cell strainer (Corning), with undigested tissue and epithelial cells being removed at each of the steps. The cell suspension was washed twice with phosphate-buffered saline (PBS) (Life technologies) by centrifugation at 500 ×g for 10 min. Finally, the cell pellet was resuspended in complete growth medium containing Dulbecco modified essential medium low glucose (DMEM-LG) (Life technologies) + 10% MSC certified fetal calf serum (FCS) (Life technologies) + 1% antibiotic and antimycotic (Life technologies). Viable cells were counted in 1% eosin (Merck KGaA, Darmstadt, Germany) and cultured at 4000 cells/cm^2^ in tissue culture flasks at 37°C with 5% CO_2_. After two days, the growth medium was changed and thereafter every three to four days. When the cells reached 70–90% confluency, they were trypsinised using 0.05% trypsin/EDTA (Life technologies) and cultured as described above. At passage 2, the stromal cells were cryopreserved in 10% dimethyl sulfoxide (DMSO) (Sigma) in complete growth medium. Flow cytometry showed that they were positive for stromal markers, such as CD73, CD90, and CD105 (data not shown). To ensure that we were working with a pure population of cells, ESC_endo_ and ESC_cyst_ were used at passages three to six, as earlier passages may be contaminated with other cell types.

### 2.3. Isolation of Allogeneic Ad-MSC from Adipose Tissue

Human adipose tissue was obtained from healthy pregnant women undergoing elective caesarean section. The tissue was digested as described above but for 60 min. The tissue digest was centrifuged at 500 ×g for 10 min at 4°C. Following centrifugation, the top layer of fat and middle layer of blood were carefully removed, with the resulting cell pellet resuspended in complete growth medium as described above. The stromal cells were cultured and cryopreserved as described above. These Ad-MSC were characterized by flow cytometry for CD73, CD90, CD105, HLA classes I and II, CD14, CD45, and CD31; formation of colonies in colony-forming units-fibroblasts; and differentiation assays into the osteogenic and adipogenic mesenchymal lineages and were found to be MSC [13]. To ensure that we were working with a pure population of cells, Ad-MSC was used at passages three to six, as earlier passages may be contaminated with other cell types.

### 2.4. Cell Coculture Setup

**Figure 1:**
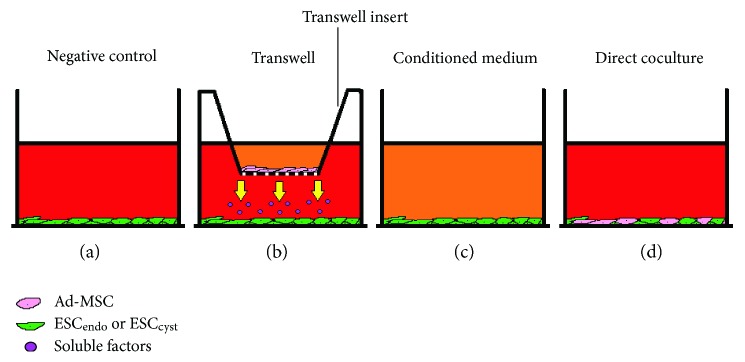
Schematic image showing the various cell coculture systems employed in the study. ESC_endo_ and ESC_cyst_ were cultured alone as a negative control (Neg C), with Ad-MSC using 0.4 *μ*m pore inserts as a transwell system in conditioned medium (Cond) derived from Ad-MSC, or with Ad-MSC in direct cell coculture (Direct).

Ad-MSC were investigated for their effects on ESC_endo_ and ECS_cyst_ using cell proliferation, apoptosis, adhesion, migration, and invasion assays. Based on optimization experiments a 1 : 1 ratio of Ad-MSC to ESC_endo_ and ESC_cyst_ was selected for the transwell and direct cell coculture experiments (data not shown); the number of cells used were optimized to be within the optimum capacity of the inserts (an insert can hold up to 1.12 × 10^5^ cells) and the bottom wells (a well can hold up to 3.8 × 10^5^ cells) according to Corning. The different cell coculture systems that were employed in the study are shown in Figure 1; each of the cell coculture systems mimic the effects MSC may potentially be causing *in vivo*, and therefore, they are all representative and hence essential to give an overall picture of the potential effects of MSC.

In the eosin exclusion assay, unprimed Ad-MSC and Ad-MSC primed with interferon gamma (IFN-*γ*) (100 U/mL, Sigma) or IFN-*γ* (100 U/mL) + tumor necrosis factor alpha (TNF-*α*) (10 ng/mL, PeproTech, London, UK) and their conditioned medium were used. MSC can be primed using proinflammatory cytokines such as IFN-*γ* and TNF-*α*, to make them more immunosuppressive and secrete more immunosuppressive factors, which means that they may be more therapeutically effective for a disease with an inflammatory basis such as endometriosis [14, 15]. Therefore, we examined if priming Ad-MSC to become more immunosuppressive would give an effect that would be different to unprimed MSC. For the carboxyfluorescein succinimidyl ester (CFSE), MTT (3-(4,5-dimethylthiazol-2-yl)-2,5-diphenyltetrazolium bromide), apoptosis, adhesion, tube formation, migration, and invasion assays, only unprimed Ad-MSC were used. This is because primed Ad-MSC had no effect on the proliferation of ESC_endo_ and ESC_cyst_ compared to unprimed Ad-MSC.

The following cell coculture setup was used, with slight modifications for each of the assays. When Ad-MSC were ~70% confluent, the growth medium was removed, the cells were washed twice with PBS and growth medium alone, or growth medium with IFN-*γ*, or growth medium with IFN-*γ* + TNF-*α* were added. After three days, the conditioned medium was collected, Ad-MSC harvested, and were irradiated at 20 Gy to inhibit their proliferation. The conditioned medium was centrifuged at 500 ×g for 10 min to remove cellular debris, aliquoted, and frozen at −80°C. ESC_endo_ and ESC_cyst_ were harvested and added to 12-well plates at 6000 cells/cm^2^. An equal amount of irradiated Ad-MSC was added to transwell inserts with a 0.4 *μ*m pore size (Corning) and placed in the wells with ESC_endo_ or ESC_cyst_ for direct cell coculture. Conditioned medium from Ad-MSC was also used, and untreated ESC_endo_ or ESC_cyst_ were used as controls. After 3 days of cell culture, the proliferation, apoptosis, adhesion, migration, or invasion of ESC_endo_ and ESC_cyst_ were quantified as described below.

### 2.5. Cell Proliferation Assays

Cell proliferation was measured using three different methods in order to confirm the data: the manual eosin exclusion, CFSE, and MTT assays. For the eosin exclusion assay, the total number of cells were counted using 1% eosin.

For the CFSE assay, ESC_endo_ and ESC_cyst_ were stained with 1 *μ*M CFSE (Life Technologies) for 10 min at 37°C with 5% CO_2_ as described previously [16], before they were added in the 12-well plates as above. The cells were incubated with 5 mL of complete growth medium for 10 min at 37°C with 5% CO_2_ to quench the reaction and to remove the remaining free dye. The cells were washed three times, resuspended in complete growth medium, and kept for 10 min at 37°C with 5% CO_2_ to allow the CFSE stain to undergo acetate hydrolysis. On day 0, CFSE-stained ESC_endo_ and ESC_cyst_ were used to set voltages on the BD FACSCalibur (Becton-Dickinson, New Jersey, United States) to ensure the cells were on the far right of the CFSE histograms. After 3 days of cell culture, ESC_endo_ and ESC_cyst_ were harvested and analyzed on a BD FACSCalibur. As described previously, the data was analyzed using the median fluorescence intensity (MFI) with the software FlowJo (Tree Star version 10.1r5 Inc., Ashland, United States), with a lower MFI representing greater cell proliferation and dilution of the CFSE dye [17]. For direct cell coculture, gating was only on the CFSE-positive ESC_endo_ and ESC_cyst_. The results are shown relative to the untreated controls (ESC alone).

For the MTT assay, after 3 days of cell culture, the growth medium was removed, centrifuged at 500 ×g for 10 min to remove cellular debris, aliquoted, and frozen at −80°C, for later use in the tube formation assay (see below). Then, ESC_endo_ and ESC_cyst_ were stained with 0.5 mg/mL MTT reagent (Life Technologies) for 4 hours at 37°C with 5% CO_2_. Afterwards, the MTT reagent was removed, the MTT crystals were solubilized in dimethyl sulfoxide (DMSO), and the plates were kept at 37°C with 5% CO_2_ for 10 min. Then, the absorbance was measured at 540 nm using the infinite F200 Pro Tecan spectrophotometer (Tecan, Mannedorf, Switzerland), with DMSO used as a blank. The absorbance for irradiated Ad-MSC cultured alone was subtracted from the absorbance of the direct cell coculture wells to account for the absorbance of ESC_endo_ and ESC_cyst_ only.

### 2.6. Analysis of Apoptosis with Annexin V Assay

The annexin V assay was used to analyze apoptosis, as previously described [18], of ESC_endo_ and ESC_cyst_, which were stained with CFSE as described above before they were added to 12-well plates and cultured with irradiated Ad-MSC. After 3 days of cell culture, ESC_endo_ and ESC_cyst_ were harvested and resuspended in 100 *μ*L annexin V binding buffer (10 mM of 4-(2-hydroxyethyl)-1-piperazineethanesulfonic acid (Life technologies) + 140 mM of sodium chloride (Sigma) + 2.5 mM of calcium chloride (Sigma)). Then, the cells were stained with annexin V PE antibody (BioLegend, California, United States) and 7-AAD (BD Biosciences, Stockholm, Sweden) for 15 min at room temperature (RT) in the dark. Then, 400 *μ*L annexin V binding buffer was added and the CFSE positive cells were analyzed on a BD LSR Fortessa (Becton-Dickinson). For direct cell coculture, gating was only on the CFSE-positive ESC_endo_ and ESC_cyst_. The data was analyzed using the software FlowJo.

### 2.7. Cell Adhesion Assay

Tissue culture-treated 48-well plates (Corning) were prepared by coating overnight with 10 *μ*g/mL human fibronectin (BD Biosciences) at 4°C, and the cell adhesion assay was carried out as previously described [19]. The remaining sites were blocked with 0.1% bovine serum albumin (Sigma) for 2 hours at RT and washed once with PBS. The 48-well plates were dried and wrapped in parafilm and stored at 4°C until use for the cell adhesion assay.

After 3 days of cell culture as described above, ESC_endo_ and ESC_cyst_ were harvested and counted. The fibronectin-coated plates were brought to RT for at least 10 min. ESC_endo_ and ESC_cyst_ were resuspended in serum-free DMDM-LG medium and seeded at 21000 cells/cm^2^. Following 2 hours of cell adhesion at 37°C with 5% CO_2_, the medium was gently removed, and the wells were washed with PBS containing 2 mM calcium chloride and 2 mM magnesium chloride. The remaining adherent cells were quantified using the MTT assay as described above.

### 2.8. Tube Formation Assay

The tube formation assay was carried out as previously described [20]. HUVEC were kindly provided by Dr. Nina Heldring (Karolinska Institutet). They were isolated (*n* = 2) as previously described [21] and expanded on 0.1% gelatin- (Kodak) coated surfaces in complete HUVEC growth medium containing human endothelial serum-free basal medium (Fisher Scientific, Göteborg, Sweden) + 10% FCS (Life technologies) + 1% penicillin and streptomycin (Life technologies). They were used for experiments at passages two to five. For the tube formation assay, all pipette tips and plates were prechilled at −20°C. Matrigel (Corning) was thawed overnight at 4°C on ice, aliquoted at 50 *μ*L per well in 96-well plates, and kept at 37°C with 5% CO_2_ for 1 hour in order for the matrigel to gel. HUVEC were harvested and counted with eosin as described above, then 20 × 10^3^ were resuspended in 50% complete HUVEC growth medium + 50% conditioned medium collected from the MTT assay (see above) and added gently on the matrigel. The cells were kept at 37°C with 5% CO_2_ for 17-18 hours, then they were visualized using the Olympus CKX41 inverted microscope (Olympus, Tokyo, Japan), and images were taken at 4x magnification capturing the whole well in the 96-well plates. The number of tubes formed per well were quantified by using the angiogenesis analyzer plugin on ImageJ (Version 1.48, National Institutes of Health, Bethesda, United States) as described previously [22].

### 2.9. Cell Migration Assay

The transwell cell migration assay was carried out as previously described [23]. After 3 days of cell culture as described above, ESC_endo_ and ESC_cyst_ were harvested and counted. ESC_endo_ and ESC_cyst_ were added at 25 × 10^3^ per transwell insert (8 *μ*m in pore diameter, Corning) in serum-free DMEM-LG growth medium in 24-well plates and were allowed to migrate towards 10% FCS in DMEM-LG growth medium at 37°C with 5% CO_2_. The negative controls were untreated ESC_endo_ and ESC_cyst_. After 20 hours, the nonmigrated cells on top of the inserts were removed using a wet cotton swab, the inserts washed with PBS, and the cells fixed with ice cold methanol for 5 minutes. The inserts were again washed with PBS, before being stained with 1% eosin for 1 hour. Finally, the inserts were washed in milliQ water and dried and 5 random fields per insert of the migrated cells were captured at 10x magnification. The number of cells that migrated were analyzed and counted using ImageJ.

### 2.10. Cell Invasion Assay

The transwell cell invasion assay was carried out as previously described [23]. After 3 days of cell culture as described above, ESC_endo_ and ESC_cyst_ were harvested and counted. On the same day, transwell inserts (8 *μ*m in pore diameter, Corning) fitting 24-well plates were coated with 0.1 mg/mL matrigel for two hours. ESC_endo_ and ESC_cyst_ were added at 25 × 10^3^ per insert in serum-free DMEM-LG growth medium, placed in the 24-well plates and allowed to invade through matrigel towards 10% FCS in DMEM-LG growth medium at 37°C with 5% CO_2_. The negative controls were untreated ESC_endo_ or ESC_cyst_. After 20 hours, the invaded cells were stained and quantified as described above for the migration assay.

### 2.11. Statistical Analysis

All statistical analyses were performed using GraphPad prism 6. When data was normally distributed, the means were analyzed with Student's *t*-test, and when it was not normally distributed, the medians were analyzed with the Mann–Whitney test. All values are shown as the mean ± standard deviations (SD). For the study, *n* refers to the number of biological replicates. Results were considered to be statistically significant if *P* < 0.05.

## 3. Results

### 3.1. Ad-MSC Increased Proliferation of Stromal Cells

To study the effects of Ad-MSC on stromal cell proliferation, Ad-MSC were cocultured with ESC directly or in a transwell system, or the effects of conditioned medium from Ad-MSC (either primed or unprimed with IFN-*γ* or IFN-*γ* + TNF-*α*) on stromal cells was examined. Cell proliferation was measured by the manual cell count, CFSE, and MTT assays.

**Figure 2:**
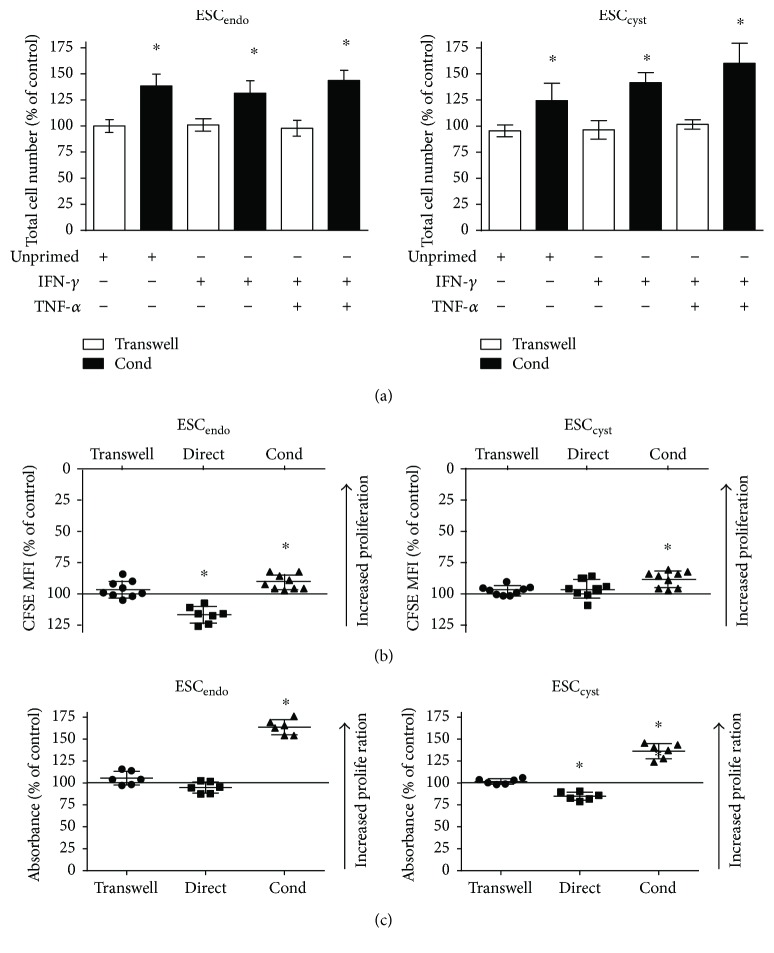
Ad-MSC increased proliferation of ESC_endo_ and ESC_cyst_. A transwell system using Ad-MSC and conditioned medium (Cond) from allogeneic unprimed and primed (100 U/mL IFN-*γ* or 100 U/mL IFN-*γ* + 10 ng/mL TNF-*α*) Ad-MSC were used in coculture with ESC_endo_ or ESC_cyst_. Direct cell coculture of unprimed Ad-MSC and ESC_endo_ or ESC_cyst_ was also carried out. Proliferation of ESC_endo_ and ESC_cyst_ was determined using the eosin exclusion (a), CFSE (b), and MTT (c) assays. Conditioned medium increased proliferation of ESC_endo_ and ESC_cyst_ (^∗^*P* < 0.05), and the transwell system had no effect on the proliferation of both cell types. The effect of direct cell coculture was more ambiguous. The data was normalized to untreated controls for each cell type. Thirty-six independent experiments (*n* = 3-4 biological replicates) were carried out in duplicates (mean ± SD).

Manual cell count showed that both unprimed and primed conditioned medium from Ad-MSC increased proliferation significantly (*P* < 0.05) for ESC_endo_ and ESC_cyst_ compared to the untreated controls (Figure 2(a)). Moreover, the CFSE and MTT assays also showed that conditioned medium from unprimed Ad-MSC increased proliferation significantly (*P* < 0.05) for ESC_endo_ and ESC_cyst_ compared to the untreated controls (Figures 2(b) and 2(c)). However, in the transwell system, there was no effect on the proliferation of ESC_endo_ and ESC_cyst_ using manual cell count, CFSE, or the MTT assays (Figure 2). As measured by the CFSE assay, direct cell coculture decreased proliferation significantly (*P* < 0.05) for ESC_endo_ but had no effect on ESC_cyst_ (Figure 2(b)). On the contrary, using the MTT assay, the direct cell coculture system significantly (*P* < 0.05) decreased proliferation for ESC_cyst_ but had no effect on ESC_endo_ (Figure 2(c)). Priming Ad-MSC with the proinflammatory cytokines IFN-*γ* and TNF-*α* resulted in no difference on cell proliferation compared to unprimed Ad-MSC, and therefore, priming was discontinued for the rest of the study. Taken together, the results showed that conditioned medium from Ad-MSC increased proliferation of ESC, and the transwell system had no effect on the proliferation of ESC. The effect of direct cell coculture was more ambiguous.

### 3.2. Ad-MSC Increased Survival of Stromal Cells

**Figure 3:**
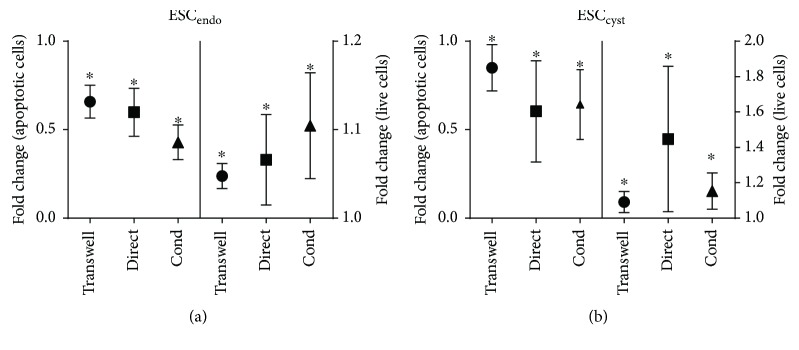
Ad-MSC reduced apoptosis and increased survival of ESC_endo_ and ESC_cyst_. A transwell system using Ad-MSC and conditioned medium (Cond) from allogeneic unprimed Ad-MSC were used in coculture with ESC_endo_ or ESC_cyst_. Direct cell coculture of unprimed Ad-MSC and ESC_endo_ or ESC_cyst_ was also carried out. Apoptosis and survival of ESC_endo_ and ESC_cyst_ was determined using the annexin V assay by flow cytometry. All three systems reduced apoptosis and increased survival of ESC_endo_ and ESC_cyst_ (^∗^*P* < 0.05). The data was normalized to untreated controls for each cell type. Four independent experiments (*n* = 4 biological replicates) were carried out in triplicates (mean ± SD).

To examine the effects of Ad-MSC on the survival and apoptosis of ESC_endo_ and ESC_cyst_, the annexin V assay using flow cytometry was used. The transwell system, conditioned medium, and direct cell coculture significantly (*P* < 0.05) reduced apoptosis and increased survival for both ESC_endo_ and ESC_cyst_ compared to the untreated controls (Figure 3).

### 3.3. Ad-MSC Did Not Increase Adhesion of ESC_cyst_

**Figure 4:**
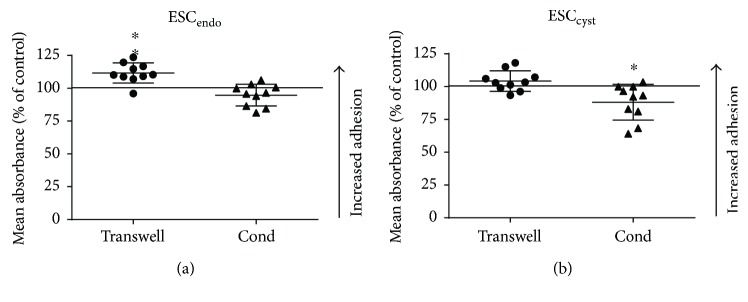
Ad-MSC did not increase adhesion of ESC_cyst_. A transwell system using Ad-MSC and conditioned medium (Cond) from allogeneic Ad-MSC were used in coculture with ESC_endo_ or ESC_cyst_. Adhesion of ESC_endo_ and ESC_cyst_ on fibronectin was determined by quantifying the number of adherent cells using the MTT assay. The transwell system increased adhesion of ESC_endo_ (^∗^*P* < 0.05), and conditioned medium reduced adhesion of ESC_cyst_ (^∗^*P* < 0.05). The data was normalized to untreated controls for each cell type. Five independent experiments (*n* = 4 biological replicates) were carried out in duplicates (mean ± SD).

To examine the effects of Ad-MSC on the adhesion of ESC_endo_ and ESC_cyst_, a fibronectin adhesion assay was employed. The transwell system increased adhesion significantly (*P* < 0.05) for ESC_endo_ but had no effect on ESC_cyst_ compared to the untreated controls (Figure 4(a)). In contrast, the conditioned medium system decreased adhesion significantly (*P* < 0.05) for ESC_cyst_ but had no effect on ESC_endo_ compared to the untreated controls (Figure 4(b)). Therefore, it can be concluded that the adhesion of ESC_cyst_ is not increased following treatment with Ad-MSC. Moreover, although the conditioned medium reduced the adhesion of ESC_cyst_, the transwell system maintained the adhesion of ESC_cyst_, which does not support our assumption that Ad-MSC may be therapeutically useful for endometriosis.

### 3.4. Ad-MSC Increased Tube Formation

**Figure 5:**
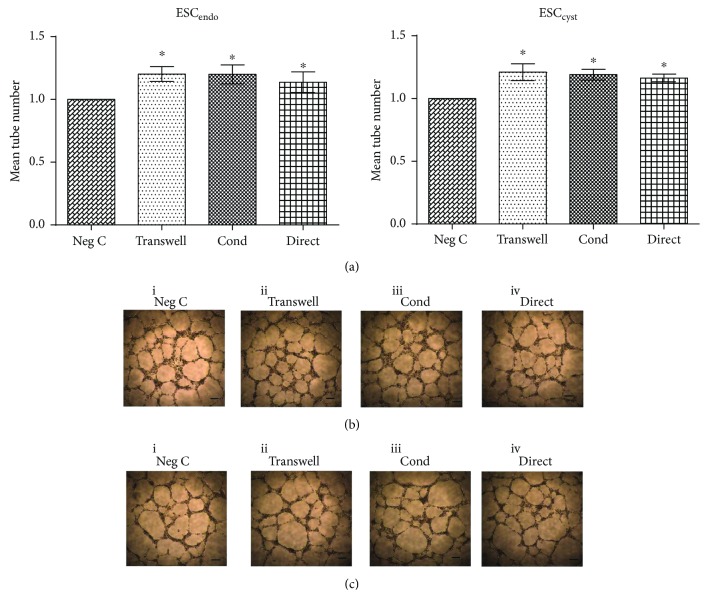
Conditioned medium from Ad-MSC/ESC_endo_ and Ad-MSC/ESC_cyst_ cocultures increased tube formation of HUVEC on matrigel. HUVEC were cultured on matrigel for 17-18 hours in conditioned medium (Cond) from cell cocultures of allogeneic Ad-MSC/ESC_endo_ or Ad-MSC/ESC_cyst_, and tube formation was determined. All conditions induced tube formation (a) (^∗^*P* < 0.05). Representative images showing connections between HUVEC forming ring or tube structures for each condition for ESC_endo_ (b) and for ESC_cyst_ (c), respectively. The images are at 4x magnification to image and quantify the whole 96-plate well. The data was normalized to untreated controls for each cell type. Four independent experiments (*n* = 4 biological replicates) were carried out in duplicates (mean ± SD). Scale bars represent 50 *μ*m at 4x magnification.

To evaluate the influence of Ad-MSC on tube formation of HUVEC, the effects of conditioned medium collected from the Ad-MSC/ESC_endo_ and Ad-MSC/ESC_cyst_ cocultures on HUVEC tube formation was studied. Conditioned medium from all three systems induced a significant (*P* < 0.05) increase in tube formation compared to the untreated controls for both ESC_endo_ and ESC_cyst_, respectively (Figure 5(a)). This shows that conditioned medium derived from cocultures of Ad-MSC and ESC can support tube formation of HUVEC *in vitro*.

### 3.5. Ad-MSC May Promote Migration of ESC_cyst_

**Figure 6:**
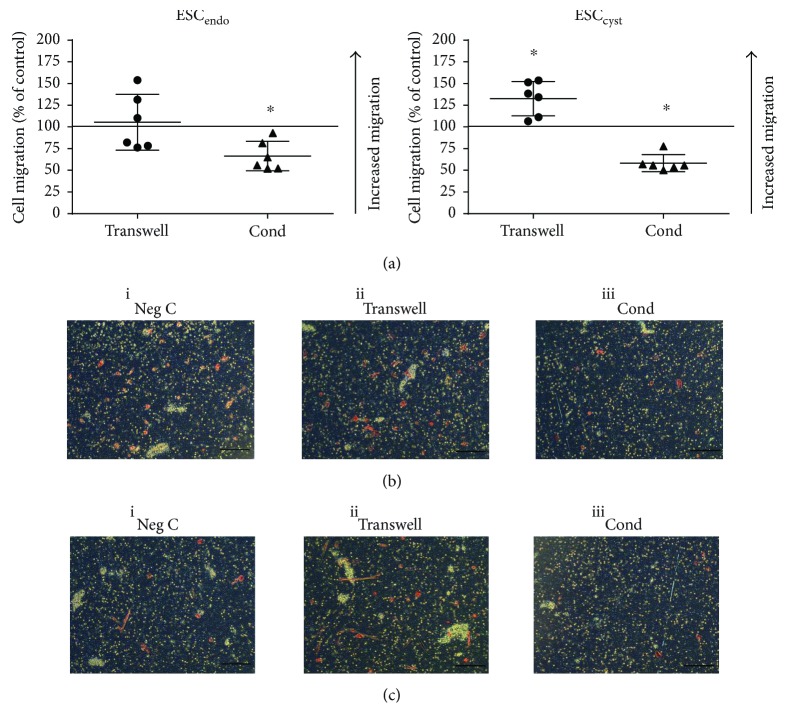
Ad-MSC may promote migration of ESC_cyst_. A transwell system using Ad-MSC and conditioned medium (Cond) from allogeneic Ad-MSC were used in coculture with ESC_endo_ or ESC_cyst_. Following cell coculture, migration of ESC_endo_ and ESC_cyst_ was determined after 20 hours using the transwell migration assay. Conditioned medium reduced migration of ESC_endo_ (^∗^*P* < 0.05). The transwell system increased migration, and conditioned medium reduced migration of ESC_cyst_ (^∗^*P* < 0.05). Representative images of migration for each condition for ESC_endo_ (b) and ESC_cyst_ (c). The 0.4 *μ*m pores of the inserts are the white dots, and the migrated cells are light/dark red in color (10x magnification). The data was normalized to untreated controls for each cell type. Three independent experiments (*n* = 3 biological replicates) were carried out in duplicates (mean ± SD).

To investigate the migratory activity of ESC, they were allowed to migrate towards 10% FCS through 8 *μ*m pore filters after being treated with conditioned medium or in a transwell system with Ad-MSC. The transwell system increased ESC_cyst_ migration significantly (*P* < 0.05) but had no effect on ESC_endo_ compared to the untreated controls (Figure 6(a), i). Moreover, the conditioned medium system reduced migration of both ESC_endo_ and ESC_cyst_ significantly (*P* < 0.05) compared to the untreated controls (Figure 6(a), ii). The results on the effects of Ad-MSC on ESC_cyst_ migration were conflicting; however, it may be concluded that Ad-MSC may promote migration of ESC_cyst_.

### 3.6. Ad-MSC Did Not Increase Invasion of ESC_endo_ and ESC_cyst_

**Figure 7:**
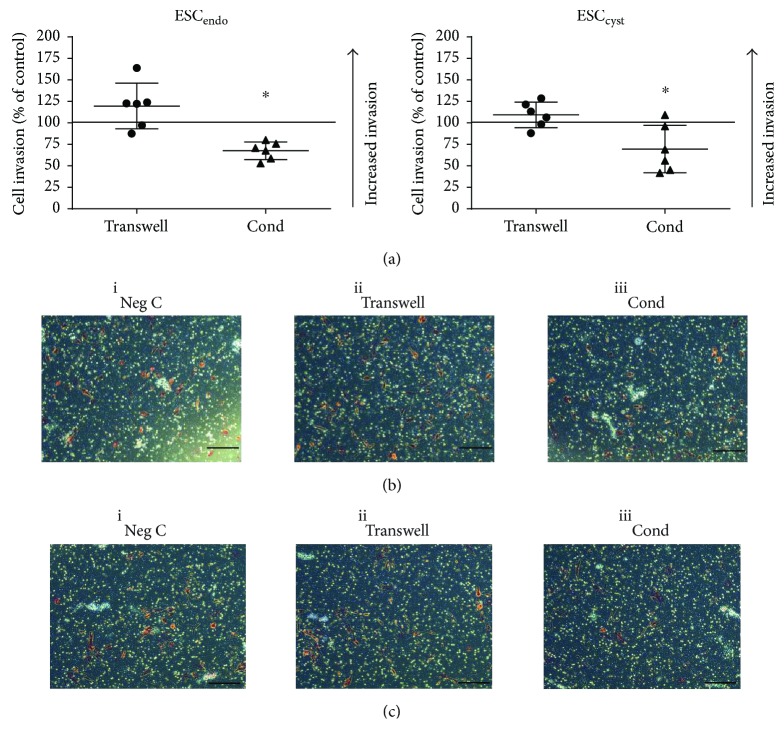
Ad-MSC did not increase invasion of ESC_endo_ and ESC_cyst_. A transwell system using Ad-MSC and conditioned medium (Cond) from allogeneic Ad-MSC were used in coculture with ESC_endo_ or ESC_cyst_. Following cell coculture, invasion of ESC_endo_ and ESC_cyst_ was determined using the transwell invasion assay after 20 hours. Conditioned medium reduced invasion of ESC_endo_ and ESC_cyst_ (^∗^*P* < 0.05). Representative images of invasion for each condition for ESC_endo_ (b) and ESC_cyst_ (c). The 0.4 *μ*m pores of the inserts are the white dots, and the invaded cells are light/dark red in color (10x magnification). The data was normalized to untreated controls for each cell type. Three independent experiments (*n* = 3 biological replicates) were carried out in duplicates (mean ± SD).

To determine the invasive capacity of ESC_endo_ and ESC_cyst_, invasion was analyzed using a matrigel transwell assay. The transwell system had no effect on the invasive capacity of ESC_endo_ and ESC_cyst_ compared to the untreated controls (Figure 7(a), i). Contrary to this, conditioned medium reduced invasion of both ESC_endo_ and ESC_cyst_ significantly (*P* < 0.05) compared to the untreated controls (Figure 7(a), ii). Therefore, it can be concluded that the invasion of ESC_endo_ and ESC_cyst_ is not increased following treatment with Ad-MSC. Moreover, although the conditioned medium reduced the invasion of ESC_endo_ and ESC_cyst_, the transwell system maintained the invasion of both cell types, which does not support our assumption that Ad-MSC may be therapeutically useful for endometriosis.

## 4. Discussion

In this study, we show that allogeneic Ad-MSC may promote ESC_cyst_ proliferation, survival, and migration, and may support ESC_cyst_ to increase tube formation of HUVEC but did not increase adhesion or invasion of ESC_cyst_* in vitro*. The effects of Ad-MSC on ESC_cyst_ shown here suggest that they should not be considered as a potential therapy for endometriosis, because they may support the pathology of endometriosis by maintaining and increasing growth of ectopic endometrial tissue. Moreover, since MSC are present in ectopic lesions in endometriosis as confirmed by us [11] and others [12], this indicates that MSC are likely involved in the pathogenesis of endometriosis.

Li et al. studied the effect of conditioned medium from MSC on ESC_endo_ and ESC_cyst_, and similar to our data, they found that MSC induce a significant increase in ESC proliferation [24]. Also, MSC isolated from Wharton's jelly induced a significant increase in ESC_endo_ proliferation in a transwell system [25]. In contrast, Xu et al. reported that umbilical cord-MSC (UC-MSC) significantly reduce proliferation of ESC_cyst_ [26]. These discrepancies between the studies may be explained by the different tissue sources for derivation of MSC, which has previously been described [27]. There are no other studies examining the effects of MSC on ESC in terms of proliferation. However, MSC have been previously shown to increase the cell proliferation of other cell types through their release of cytokines, and growth factors [28–30]. In our study, conditioned medium induced proliferation significantly of both ESC_endo_ and ESC_cyst_. In contrast, the transwell system had no effect on the proliferation of ESC_endo_ and ESC_cyst_. In the transwell system, there is a paracrine effect between ESC and Ad-MSC; Ad-MSC may become modulated by factors secreted by ESC, subsequently hindering their growth-promoting effects on ESC. Paracrine signaling is the local cell-to-cell communication through the paracrine factors (e.g., cytokines, hormones, and microvesicles) that are secreted into the extracellular environment [31]. This feedback effect is absent in the conditioned medium, which contains factors secreted by unmodulated Ad-MSC. This may explain the different results from the transwell, and the conditioned medium systems in this study. Moreover, it is unlikely that the conditioned medium effect was nonspecific; instead, it is most likely specific for Ad-MSC since the effects with conditioned medium differ from the untreated controls. The direct cell coculture system had no effect, or caused a significant decrease in the proliferation of ESC_endo_ and ESC_cyst_. The decrease in cell proliferation could be due to the growth inhibitory effects of MSC through direct contact. As previously shown, MSC mediate their greatest inhibition on cell proliferation *in vitro* through direct contact compared to the transwell system [32].

In this study, Ad-MSC significantly decreased apoptosis, and increased survival of ESC_endo_ and ESC_cyst_, which is in keeping with studies in other cell types [33–35]. These results suggest that Ad-MSC may decrease apoptosis, increase survival of ESC_endo_ and ESC_cyst_, and may support endometriosis. In contrast, UC-MSC have been reported to induce apoptosis of ESC_cyst_ in a transwell system by a mechanism involving the tensin homologue gene (PTEN), an important housekeeping gene in endometrial tissue [26]. This discrepancy to our study may be due to the use of a different source of MSC [27].

Cell adhesion is crucial in the development of endometriosis to allow the attachment of endometrial tissue onto the mesothelial lining in the pelvic cavity following retrograde menstruation. Treatment via the transwell system was found to cause a significant increase in the adhesion of ESC_endo_, but it had no effect on the adhesion of ESC_cyst_. Conditioned medium caused a significant decrease in the adhesion of ESC_cyst_, but it had no effect on the adhesion of ESC_endo_. These results can be understood in light of the results of cell proliferation, since it is known that cells that divide rapidly are likely to be less adherent [36]. Moreover, the results may be explained by differences between the transwell and the conditioned medium systems; there is a paracrine effect in the transwell system that is absent in the conditioned medium as described above. To our knowledge, there are no other studies in the literature that have examined the effects of MSC on the adhesion of ESC_endo_ and ESC_cyst_.

The tube formation assay is commonly used to quantify the effects of various treatments on the ability of HUVEC to form tubes on a gelled membrane matrix as an *in vitro* model of angiogenesis [20]. Conditioned medium collected from Ad-MSC/ESC_endo_ and Ad-MSC/ESC_cyst_ cell cocultures significantly induced tube formation of HUVEC compared to untreated controls. There are no previous studies that have examined the outcome of MSC-treated ESC_endo_ and ESC_cyst_ on HUVEC tube formation. Our results are in line with previous reports showing that MSC have the ability to increase tube formation [37–39].

The transwell system significantly increased the migration of ESC_cyst_ compared to the untreated control but had no effect on ESC_endo_. Moreover, the transwell system had no effect on the invasion of either cell type. Meanwhile, the conditioned medium system significantly decreased the migration and invasion of both ESC_endo_ and ESC_cyst_. Migration and invasion require initial cell adherence, and therefore, these results are in line with the cell adhesion data and may be explained by differences between the transwell and the conditioned medium systems [40]. The transwell system has a paracrine effect that is absent in the conditioned medium, as described above. Only one previous study has examined the effect of MSC conditioned medium on the migration and invasion of ESC_endo_ and ESC_cyst_ [24]. In contrast to our results, they found that migration and invasion of both cell types were significantly increased compared to untreated controls [24]. Again, this discrepancy could be explained by the different sources of MSC [27]. Their study used MSC isolated from endometriotic ovarian cysts and the endometrium of women with endometriosis, and we used allogeneic Ad-MSC [24].

The limited number of donors and the donor hormonal status did not affect the consistency of data acquired from the *in vitro* cell experiments. Statistically significant results were observed and meaningful conclusions could still be drawn. Moreover, a similar number of patients have been used in other studies [10, 41]. Also, the use of Ad-MSC isolated from the adipose tissue of pregnant women may not be optimal; however, it has been previously shown that pregnancy has no detrimental effects on the nature of the isolated Ad-MSC [42]. Nevertheless, it must be remembered that this is an *in vitro* study, and additional *in vitro* and *in vivo* studies are needed to validate the findings of the current study. The present study suggests that allogeneic Ad-MSC should not be used as a potential therapy for endometriosis, because they may support the pathology of endometriosis by maintaining and increasing growth of ectopic endometrial tissue. In addition, since MSC are present in ectopic lesions in endometriosis, this means they are likely involved in the pathogenesis of endometriosis. This is the most extensive *in vitro* study showing that this may indeed be true, and will be significant in further understanding the pathogenesis of endometriosis to potentially find new therapeutics by targeting MSC.

## 5. Conclusion

In conclusion, Ad-MSC should not be considered as a potential therapy for endometriosis with endometriotic ovarian cysts, because they may promote proliferation, survival, and migration of ESC_cyst_ and support ESC_cyst_ to promote angiogenesis of endothelial cells to worsen the pathology. However, further studies examining other sources of MSC are needed to confirm if MSC are indeed an ineffective therapy for endometriosis.
